# A phospho-proteomic screen identifies substrates of the checkpoint kinase Chk1

**DOI:** 10.1186/gb-2011-12-8-r78

**Published:** 2011-08-18

**Authors:** Melanie Blasius, Josep V Forment, Neha Thakkar, Sebastian A Wagner, Chunaram Choudhary, Stephen P Jackson

**Affiliations:** 1 The Gurdon Institute and Department of Biochemistry, University of Cambridge, Tennis Court Road, Cambridge CB2 1QN, UK; 2 NNF Center for Protein Research, Faculty of Health Sciences, University of Copenhagen, Blegdamsvej 3B, Copenhagen DK-2200, Denmark

## Abstract

**Background:**

The cell-cycle checkpoint kinase Chk1 is essential in mammalian cells due to its roles in controlling processes such as DNA replication, mitosis and DNA-damage responses. Despite its paramount importance, how Chk1 controls these functions remains unclear, mainly because very few Chk1 substrates have hitherto been identified.

**Results:**

Here, we combine a chemical genetics approach with high-resolution mass spectrometry to identify novel Chk1 substrates and their phosphorylation sites. The list of targets produced reveals the potential impact of Chk1 function not only on processes where Chk1 was already known to be involved, but also on other key cellular events such as transcription, RNA splicing and cell fate determination. In addition, we validate and explore the phosphorylation of transcriptional co-repressor KAP1 Ser473 as a novel DNA-damage-induced Chk1 site.

**Conclusions:**

By providing a substantial set of potential Chk1 substrates, we present opportunities for studying unanticipated functions for Chk1 in controlling a wide range of cellular processes. We also refine the Chk1 consensus sequence, facilitating the future prediction of Chk1 target sites. In addition, our identification of KAP1 Ser473 phosphorylation as a robust readout for Chk1 activity could be used to explore the *in vivo *effects of Chk1 inhibitors that are being developed for clinical evaluation.

## Background

Protein phosphorylation is an abundant post-translational modification that plays crucial roles in essentially all cellular processes, including the DNA-damage response (DDR). Key aspects of the DDR are the slowing or stopping of cell cycle progression by DNA-damage checkpoint pathways, which in part operate to allow time for DNA repair to take place, and the induction of apoptosis if the damage is too severe. The main DNA-damage signaling pathways are initiated by the DNA-damage sensor protein kinases ATM (ataxia-telangiectasia mutated) and ATR (ataxia-telangiectasia and Rad3 related). In addition to them cooperating with the related kinase DNA-PK to phosphorylate various proteins at DNA-damage sites, such as histone H2AX (to yield a phosphorylated species termed γH2AX), ATM and ATR phosphorylate and activate the downstream effector checkpoint kinases Chk2 and Chk1, respectively (for recent reviews, see [1,2]). Notably, a third checkpoint effector kinase has recently been shown to function downstream of ATM/ATR, working in parallel to Chk1 [3]. This p38MAPK/MAPKAP-K2 (MK2) complex is activated in response to DNA-damaging agents such as ultraviolet light and shares several checkpoint-relevant substrates with Chk1. The degree of overlap between Chk1, Chk2 and MK2 is not known, but it has been suggested that MK2 acts predominantly in the cytoplasm in the later phases of the DDR (reviewed in [4]). The importance of the DDR is underscored by the fact that failure to activate DNA-damage checkpoints increases genomic instability and can lead to a range of diseases [1]. For instance, people or animals with defects in the ATM/Chk2 pathway display heightened predisposition to cancer, although cells deficient in ATM or Chk2 are otherwise viable [5,6]. By contrast, ATR and Chk1 are essential for mammalian cell viability, and knockout mice for these proteins display embryonic lethality [7-10]. The essential roles of Chk1 in the cell are still unclear, mainly because very few substrates of Chk1 have been identified to date.

As hundreds of protein kinases are encoded by the human genome, all of which use ATP as their co-factor, and because tens-of-thousands of potential phosphorylation sites have been identified in human proteins [11,12], it has been challenging to define kinase-substrate relationships. Identification of such pairs is usually based on the researcher making an educated guess, followed by *in vitro *kinase assays and *in vivo *confirmation with phospho-specific antibodies. The identity of the kinase is then further confirmed by the use of specific kinase inhibitors and/or short-interfering RNA (siRNA)-mediated kinase depletion. Screening for large numbers of protein kinase substrates has proven more difficult, although recent antibody-based screens have identified hundreds of putative ATM and ATR substrates [13,14]. As such screenings require the previous identification of sites of substrate phosphorylation and corresponding antibodies that specifically recognize these phosphorylated motifs, these approaches are unfortunately not feasible for kinases such as Chk1 that have few known targets, that share phosphorylation motifs with other kinases and/or lack a highly specific target motif.

Chemical genetics employs small-molecule modulators of protein and nucleic acid activities to elucidate cellular functions of their targets. Notably, Shokat and co-workers [15] have developed a chemical-genetics system to modulate the activity of a protein kinase by mutating an amino acid residue in its ATP-binding pocket (the 'gatekeeper' residue), allowing the resulting kinase - often called an analogue-sensitive (*as*)-kinase - to accommodate a bulky ATP analogue. This modified ATP-binding pocket allows the specific inhibition of the *as*-kinase *in vivo *by using specific cell-membrane-permeable, non-hydrolysable ATP analogues. More recently, new methods to identify *in vitro *substrates of *as*-kinases have been developed that involve the use of a hydrolysable and labeled ATP analogue in cell extracts. This latter approach has been successfully applied to the identification of new substrates of protein kinases such as CDK1/CyclinB, CDK7, and CDK2/CyclinA [16-18]. Here, by applying this technique to Chk1, we identify 268 phosphorylation sites in 171 proteins, thus providing for the first time an unbiased list of putative Chk1 substrates.

## Results

### Production of an analogue-sensitive Chk1

**Figure 1:**
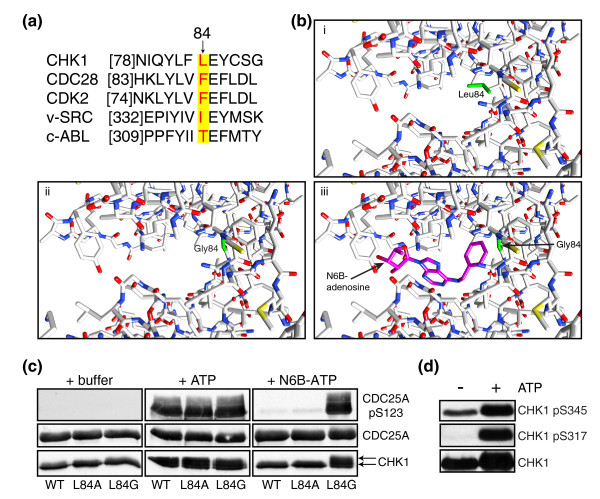
**Producing a Chk1 kinase derivative able to use N6-benzyl(N6B)-ATP**. **(a) **Amino acid alignment of ATP-binding pockets of human Chk1, *Saccharomyces cerevisiae *Cdc28, human Cdk2 and c-Abl, and viral v-Src. The identified gatekeeper amino acids are highlighted. **(b) **ATP-binding pocket of Chk1 (based on PDB entry 1IA8) showing the position of the gatekeeper residue Leu84 (i), the L84G mutation (ii), or the L84G mutated ATP-binding pocket accommodating N6B-adenosine (iii). Models were drawn by Chimera software [60]. **(c) **Chk1-L84G can use ATP analogues. *In vitro *kinase assay using wild type (WT) or gatekeeper mutant versions (L84A, L84G) of Chk1 in the presence of ATP or N6B-ATP. Active kinases phosphorylate Cdc25A on Ser123 as detected by phospho-specific antibody. Chk1 mobility shift due to autophosphorylation is indicated by arrows; 0.5 μg of each recombinant protein was used. **(d) **Recombinant WT Chk1 autophosphorylates on Ser345 and Ser317 as detected by phospho-specific antibodies; 1 μg of recombinant Chk1 was used.

Amino acid alignment of the ATP-binding region of Chk1 with those of protein kinases for which *as *versions have been already successfully generated suggested that Leu84 should behave as the gatekeeper residue (Figure 1a). Modeling ATP-analogue binding in the ATP-binding pocket of Chk1 further supported this idea, as it indicated that, while the bulky benzyl group of an ATP analogue would not fit inside the wild type Chk1 ATP-binding site, it probably could be accommodated if Leu84 was mutated to a smaller residue such as glycine (Figure 1b). Accordingly, we mutated Leu84 to alanine or glycine and then carried out *in vitro *kinase assays with these and wild type Chk1 in the presence of the known Chk1 substrate Cdc25A. Importantly, wild type and both mutated versions of Chk1 were able to use ATP, as evidenced by them mediating Cdc25A phosphorylation on Ser123 as detected by western blotting with a Ser123 phospho-specific antibody [19] (Figure 1c). By contrast, only the leucine-to-glycine gatekeeper-mutated Chk1 derivative Chk1-L84G phosphorylated Cdc25A in the presence of the ATP analogue N6-benzyl(N6B)-ATP (Figure 1c). The induction of Cdc25A phosphorylation in such assays paralleled that of Chk1 autophosphorylation, as evidenced by the appearance of a slower-migrating Chk1 band on the western blots (Figure 1c, lower panels, lanes 4 to 6 and 9). We did not characterize this Chk1 autophosphorylation further but noted that, while Chk1 is phosphorylated on Ser317 and Ser345 by ATR after DNA damage and these phosphorylations are thought to be important for Chk1 kinase activity [9,20], both Ser317 and Ser345 became phosphorylated upon incubating recombinant Chk1 in the presence of ATP (Figure 1d). Collectively, these data suggested that Chk1 autophosphorylation *in vitro *can mimic ATR activation of Chk1, and more importantly, revealed that Chk1-L84G serves as an active *as *version of Chk1.

### *as*-Chk1 identifies new *in vitro *substrates and phosphorylation sites

**Figure 2:**
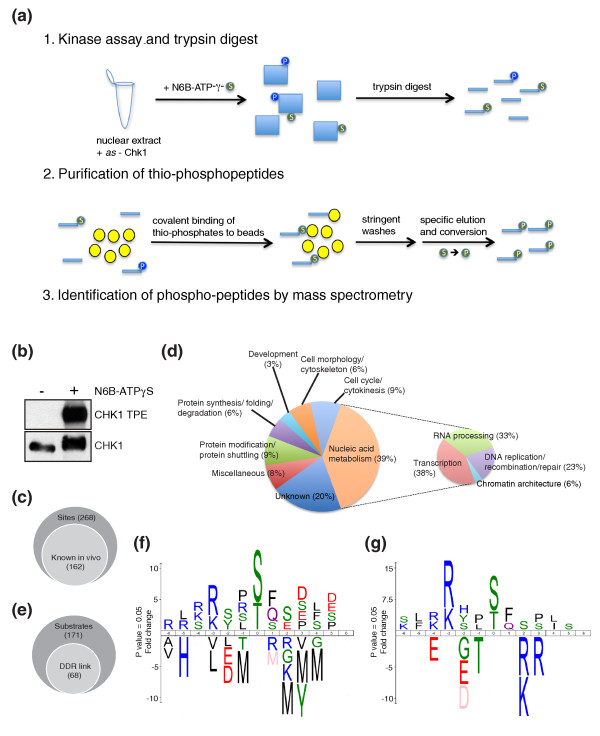
**Bioinformatic analyses of potential Chk1 substrates based on phospho-peptides identified by mass spectrometry**. **(a) **Schematic for *in vitro *labeling and identification of Chk1 substrates. **(b) ***as*-Chk1 uses N6B-ATPγS as detected by antibodies recognizing thio-phosphorylation (thio-phosphate ester (TPE) moiety). **(c) **Euler diagram depicting the proportion of phospho-sites identified known to occur *in vivo *[11,12]. **(d) **Classification of identified Chk1 substrates based on biological processes (Gene Ontology Consortium). Proteins involved in nucleic acid metabolism were further classified. **(e) **Euler diagram depicting the proportion of proteins found in this screen with links with the DNA-damage response (DDR; comparison with [13,14,21,23-25]). **(f) **Frequencies of amino acids surrounding phospho-sites identified in our screen. The x-axis represents the sequence window, with the phosphorylated residue in the middle. Amino acid size depicts fold enrichment (positive, above y-axis) or under-representation (negative, below y-axis) after normalization to amino acid occurrences in the human proteome. Amino acid colors: black, hydrophobic; blue, basic; red, acidic; green, polar; purple, ester. Residues shown in pink were never found in a given position. Note that phospho-peptides containing cysteine were not recovered due to methodological limitations [16]. Diagrams were made with IceLogo software [61]. **(g) **IceLogo for phospho-peptides with R/K at -3.

A recent, elegant method developed to identify substrates of an *as*-kinase involves the use of an ATP analogue carrying a thio-phosphate group [16]. In this approach, once the kinase reaction is performed with the *as*-kinase and its potential substrates in the presence of the ATP analogue, proteins are digested by trypsin (Figure 2a, step 1) and thio-phosphorylated peptides are specifically isolated via their specific covalent binding to iodo-acetyl agarose beads. After several stringent and extensive washes, the thio-phosphorylated peptides are then specifically eluted with an oxidizing agent that at the same time converts them into standard phospho-peptides (Figure 2a, step 2) that can subsequently be analyzed by mass spectrometry (Figure 2a, step 3). Firstly, to test whether *as*-Chk1 could also use a thio-phosphate ATP analogue (N6B-ATPγS), we carried out an *in vitro *kinase assay. Importantly, as shown in Figure 2b, *as*-Chk1 efficiently autophosphorylated in the presence of N6B-ATPγS, as revealed both by the generation of a slower-migrating, modified version of the protein and by direct detection of the auto-modified protein with an antibody specific to the thio-phosphate ester moiety.

As an approach to identify Chk1 target proteins, we next carried out a kinase assay with *as*-Chk1 and N6B-ATPγS in the presence of human HeLa cell nuclear extract. To control for the possibility of background signals arising from the hypothetical use of N6B-ATPγS by endogenous kinases, we carried out an equivalent reaction without the addition of recombinant *as*-Chk1. Both samples were then processed the same way (Figure 2a) and all phospho-sites identified in both the control reaction (without *as*-kinase) and the *as*-kinase reaction were discarded. This analysis thus produced a list of 268 phosphorylation sites in 171 proteins that were only generated in the presence of *as*-Chk1 (Additional file 1). Notably, most of the identified phosphorylation sites also occur *in vivo*, as revealed by 62% of them existing in the two protein phosphorylation databases PhosphoSite [11] and PHOSIDA [12] (Figure 2c).

As shown in Figure 2d, the proteins identified in the screen as Chk1 targets are involved in a variety of biological processes, the majority of them playing roles in nucleic acid metabolism. Further analysis of this subgroup revealed that most of the proteins are involved in either transcription or RNA processing (Figure 2d), in agreement with recent data indicating close linkages between genome stability and RNA synthesis/metabolism [21-23]. Furthermore, although our screen was not aimed specifically at identifying DNA-damage-induced phosphorylations by Chk1, almost 40% of the substrates we identified overlapped with those identified in recently published DDR-focused phospho-proteomic screens [13,14,21,23-25] (Figure 2e).

Some protein kinases target a well-defined consensus amino acid sequence, allowing the prediction of potential substrates. A clear Chk1 consensus has not been established so far due to the limited number of its known substrates, although approaches using peptide libraries for *in vitro *kinase assays have suggested a general preference for an arginine residue in the -3 position and a hydrophobic residue at -5 [26,27]. However, several exceptions to this consensus have been observed *in vitro *and *in vivo*, as is the case for Ser20 of p53 [28,29] and Thr916 of Claspin [30]. To establish target-sequence preferences for Chk1 arising in our screen, we defined the frequency values for amino acid residues surrounding the 268 identified phosphorylation sites and then normalized these values to the different frequencies of each amino acid in the human proteome. As shown in Figure 2f, this allowed us to assess, at each position relative to the phosphorylation site, whether a particular amino acid was statistically over-represented (above the central line), under-represented (below the line), or not significantly selected one way or the other (not indicated). Strikingly, this revealed that Chk1 targets arising in our screen displayed an overall bias towards the presence of basic residues amino-terminal to the phosphorylated site. While this included a strong over-representation of Arg and Lys at position -3, as previously reported [26,27], we observed little selection for hydrophobic residues at -5 (Figure 2f). Additional, albeit weaker, over-representations included those for Ser and negatively charged (Glu/Asp) residues between positions +2 and +5. Notably, in addition to our data indicating positive amino acid residue selections within the Chk1 motif, clear amino acid under-representations were also evident at certain positions (Figure 2f). Perhaps surprisingly given its partially basic character, His was not over-represented in the region amino-terminal to the phosphorylation site and was, in fact, strongly disfavored at position -5. Moreover, acidic residues were strongly disfavored at position -2, while Met was clearly disfavored at position -1. Under-representation of Met, together with other bulky, generally hydrophobic residues, was also observed carboxy-terminal to the phosphorylated residue, particularly at positions +2 to +4.

Yet further amino acid residue biases became evident when we analyzed subsets of Chk1 target sequences. A prime example of this is provided when we focused on the set of 120 Chk1-target phospho-peptides displaying a basic residue (Lys or Arg) at position -3 (Figure 2g). In this set of phospho-peptides, slight over-representations of hydrophobic residues at position -5, -1, and +4 were observed along with a slight preference for Arg/Lys residues at -4. More striking, however, was the pattern of under-represented amino acid residues, which included Thr at -1 and basic residues at +2 and +3. Also clearly under-represented were acidic residues at -2 and -4 surrounding the basic residue at -3. Intriguingly, additional differences in amino acid representation profiles were apparent when the set of Chk1 targets containing Arg/Lys at -3 was split into those containing phospho-Ser or phospho-Thr. For instance, while there was a clear enrichment of hydrophobic residues -5 to phospho-Thr, this was not the case for targets containing phospho-Ser (Additional file 2). Taken together, these results indicate that substrate sequence preferences for Chk1 are complex, with both positive and negative selections being evident. Furthermore, they indicate that, for Chk1 substrates bearing Arg/Lys at -3, the preferred consensus sequence can be denoted R/K-R/K-d/e-t-S*/T*-X-r/k-r (applying a cut-off of five-fold enrichment), where phosphorylated residues are indicated by asterisks, preferred amino acids are in capital letters, disfavored ones are in lower case and × indicates no preference.

### KAP1-Ser473 phosphorylation is DNA-damage induced

**Figure 3:**
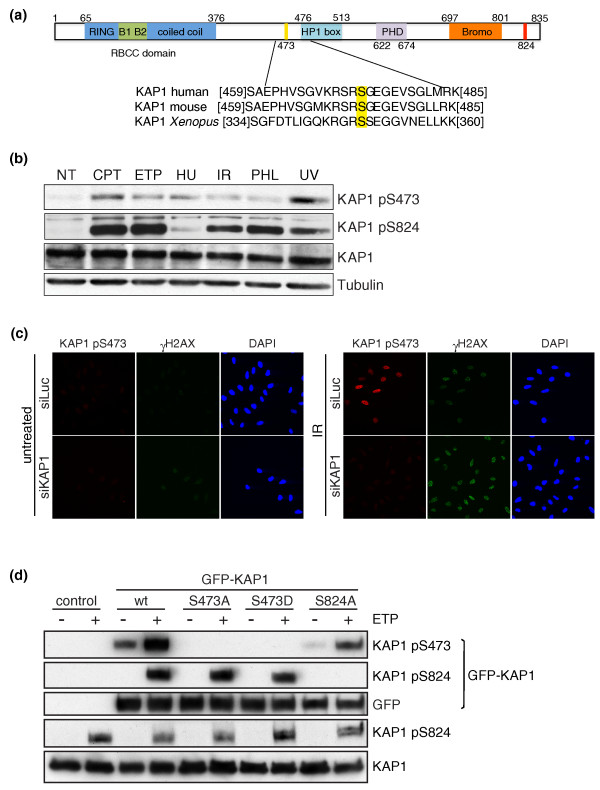
**KAP1 Ser473 phosphorylation upon DNA damage**. **(a) **Schematic of human KAP1; known domains are highlighted in color and labeled with bounding amino acid residue numbers. DNA-damage-induced Ser824 phosphorylation site is marked in red. Inset shows an alignment of the region surrounding Ser473 of human [Swissprot: Q13263], mouse [Swissprot: Q62318], and *Xenopus laevis *KAP1 [Swissprot: Q2TAS5] with the phosphorylated residue highlighted in yellow. **(b) **KAP1 phospho-Ser473 is detected on western blot after treating cells with various DNA-damaging agents. U2OS cells were not treated (NT) or treated with 1 μM camptothecin (CPT) for 2 h, 5 μM etoposide (ETP) for 2 h, 2 mM hydroxyurea (HU) for 12 h, 10 Gy of ionizing radiation (IR) 1 h before harvesting, 60 μg/ml phleomycin (PHL) for 1 h, or 10 J/m^2 ^of ultraviolet light (UV) 1 h before harvesting. **(c) **Antibodies against KAP1 phospho-Ser473 are specific in immunofluorescence. U2OS cells were transfected with siLuc or siKAP1, irradiated with 20 Gy IR and fixed 2 h afterwards. **(d) **Specificity of KAP1 phospho-Ser473 antibody by western blotting. U2OS cells stably expressing wild type (wt), S473A, S473D, or S824A versions of GFP-KAP1 were treated with 5 μM etoposide (ETP) for 4 h. Phosphorylation of endogenous KAP1 on Ser824 was used as a DNA-damage readout.

Through identifying phosphorylation sites arising from our screen that conformed well to the target motifs defined above, that were relatively conserved throughout evolution and that occurred *in vivo *as shown by their inclusion in the PhosphoSite and/or PHOSIDA databases [11,12], we derived a shortlist of Chk1 targets for further characterization (Table 1). Of these, we first focused on Ser473 of the human transcriptional co-repressor KAP1 (Krüppel-associated box domain-associated protein 1; also known as TRIM28 or Tif1β), which has previously been linked to the DDR [31]. KAP1 is an essential protein with a role in early mammalian development [32] and is phosphorylated on Ser824 by ATM in response to DNA damage [31]. This ATM-dependent phosphorylation is believed to release KAP1 from its usual chromatin-bound state, an event that triggers chromatin relaxation and promotes DNA double-strand break (DSB) repair within heterochromatin [31,33,34]. Notably, Ser473 lies just amino-terminal to the conserved heterochromatin protein 1 (HP1) box of KAP1 that mediates its interaction with the heterochromatin-associated protein HP1 (Figure 3a). Furthermore, while the motif containing human KAP1 Ser473 is not present in the KAP1-related proteins Tif1α and Tif1γ, it is well conserved in vertebrate KAP1 counterparts, including those of mouse and *Xenopus*, suggesting that it is likely to be important functionally (Figure 3a; note that, like Ser473 itself, the Arg at -3 is particularly highly conserved).

**Table 1:** Selected Chk1 substrates identified in this screen

Protein name	Gene name	Phospho-site identified
Alpha-adducin	*ADD1*	KKFRTP**S**FLKKSK (S726)
Rho guanine nucleotide exchange factor 2	*ARHGEF2*	GLRRIL**S**QSTDSL (S172)
Uncharacterized protein C10orf47	C10orf47	SSSRSR**S**FTLDDE (S43)
Calmodulin-regulated spectrin-associated protein 2	*CAMSAP1L1*	GITRSI**S**NEGLTL (S464)
Coiled-coil domain-containing protein 49	*CCDC49*	GYTRKL**S**AEELER (S337)
Cell division cycle 2-like protein kinase 5	*CDC2L5*	SRSRHS**S**ISPSTL (S437)
RNA polymerase-associated protein CTR9 homolog	*CTR9*	RPRRQR**S**DQDSDS (S1081)
Flap endonuclease 1	*FEN1*	VLMRHL**T**ASEAKK (T195)
Golgin subfamily A member 4	*GOLGA4*	LQLRVP**S**VESLFR (S71)
General transcription factor 3C polypeptide 1	*GTF3C1*	RLVRNL**S**EEGLLR (S667)
Zinc finger protein 40	*HIVEP1*	SSKRML**S**PANSLD (S1749)
Heterogeneous nuclear ribonucleoprotein M	*HNRNPM*	GMDRVG**S**EIERMG (S432)
Importin subunit alpha-2	*KPNA2*	LKRRNV**S**SFPDDA (S54)
LIM domain only protein 7	*LMO7*	IMRRGE**S**LDNLDS (S1510)
Microtubule-associated protein 4	*MAP4*	RLSRLA**T**NTSAPD (T925)
Matrin-3	*MATR3*	QLKRRR**T**EEGPTL (T150)
		FDDRGP**S**LNPVLD (S195)
Myb-binding protein 1A	*MYBBP1A*	LVIRSP**S**LLQSGA (S1310)
Probable E3 ubiquitin-protein ligase MYCBP2	*MYCBP2*	VFQRSY**S**VVASEY (S3440)
Nance-Horan syndrome protein	*NHS*	KLRRRK**T**ISGIPR (T380)
Nuclear pore complex protein Nup153	*NUP153*	DAKRIP**S**IVSSPL (S330)
O-GlcNAc transferase subunit p110	*OGT*	PTKRML**S**FQGLAE (S20)
Oxysterol-binding protein-related protein 11	*OSBPL11*	ISQRRP**S**QNAISF (S189)
PHD finger protein 8	*PHF8*	EGTRVA**S**IETGLA (S904)
Pleiotropic regulator 1	*PLRG1*	KIQRMP**S**ESAAQS (S119)
Protein phosphatase methylesterase 1	*PPME1*	HLGRLP**S**RPPLPG (S15)
Protor-1	*PROTOR1*	LLRRSR**S**GDVLAK (S240)
RNA-binding protein 7	*RBM7*	IIQRSF**S**SPENFQ (S136)
RNA-binding protein 14	*RBM14*	SDYRRL**S**ESQLSF (S618)
		SFRRSP**T**KSSLDY (T629)
Telomere-associated protein RIF1	*RIF1*	NKVRRV**S**FADPIY (S2205)
60S ribosomal protein L19	*RPL19*	PQKRLA**S**SVLRCG (S12)
Sentrin-specific protease 2	*SENP2*	LLRRKV**S**IIETKE (S344)
Paired amphipathic helix protein Sin3a	*SIN3A*	QIRRHP**T**GTTPPV (T432)
Serine/arginine repetitive matrix protein 2	*SRRM2*	QTPRPR**S**RSPSSP (S1497)
		PRPRSR**S**PSSPEL (S1499)
TBC1 domain family member 4	*TBC1D4*	VIQRHL**S**SLTDNE (S485)
		MRGRLG**S**VDSFER (S588)
Treslin (C15orf42)	*TICRR*	ALIRHK**S**IAEVSQ (S865)
		SVQRVH**S**FQQDKS (S1045)
Transcription intermediary factor 1-beta, KAP1	*TRIM28*	GVKRSR**S**GEGEVS (S473)
TRIP12 protein	*TRIP12*	GLARAA**S**KDTISN (S1078)
ATP-dependent DNA helicase 2 subunit 1, Ku70	*XRCC6*	FTYRSD**S**FENPVL (S477)
Nuclear-interacting partner of ALK	*ZC3HC1*	FFSRVE**T**FSSLKW (T84)
Zinc finger protein 395	*ZNF395*	SPVRSR**S**LSFSEP (S447)

Proteins in this list were selected based on the following criteria: (i) phosphorylation site conserved from human to *Xenopus laevis*; (ii) presence of an arginine residue at position -3 with respect to the phosphorylation site; (iii) phospho-site known to occur *in vivo *(PhosphoSite and Phosida databases [11,12]). For a detailed list of all mass spectrometry results, see Additional file 1.

To assess whether KAP1 Ser473 might be phosphorylated in response to DNA damage, we used a commercial phospho-specific antibody raised against this site (see Materials and methods). Through western immunoblot analyses, we found that KAP1 detection with this antibody was induced when cells were treated with various DNA-damaging agents, including the DNA topoisomerase I inhibitor camptothecin, the DNA topoisomerase II inhibitor etoposide, the DNA-replication inhibitor hydroxyurea, ionizing radiation (IR), the radio-mimetic drug bleomycin and ultra-violet light (Figure 3b). In addition, while this antibody only weakly stained untreated cells, exposure to IR produced pan-nuclear immunostaining in control cells but not in cells treated with siRNA directed against KAP1 (Figure 3c).

To further verify the specificity of the phospho-KAP1 Ser473 antibody, we created human U2OS cell lines stably expressing wild type KAP1, a non-phosphorylatable Ser473-to-Ala mutant (S473A) or a potential phospho-mimicking Ser473-to-Asp derivative (S473D). Importantly, while the antibody detected wild type KAP1 from cells that had been treated with etoposide, it did not detect either KAP1-S473A or KAP1-S473D after such treatment (Figure 3d). In parallel with these analyses, we assessed ATM-mediated phosphorylation of KAP1 on Ser824 and also employed a U2OS cell line stably expressing a KAP1 derivative in which Ser824 was mutated to Ala (S824A). This revealed that phosphorylations of Ser473 and Ser824 are independent events, as no difference in the phosphorylation of one site was observed when the other site was mutated (Figure 3d). Moreover, the DNA-damage induction profiles of the two sites were also markedly different, with Ser824 being mainly induced by DSB-inducing agents, while Ser473 was generated at similar levels by all DNA-damaging treatments employed, including low doses of hydroxyurea and ultraviolet light that produce few or no DSBs (Figure 3b). Collectively, these data indicated that KAP1 Ser473 is phosphorylated when cells are treated with a wide variety of DNA-damaging agents.

### KAP1 Ser-473 phosphorylation is mediated by Chk1 and Chk2

**Figure 4:**
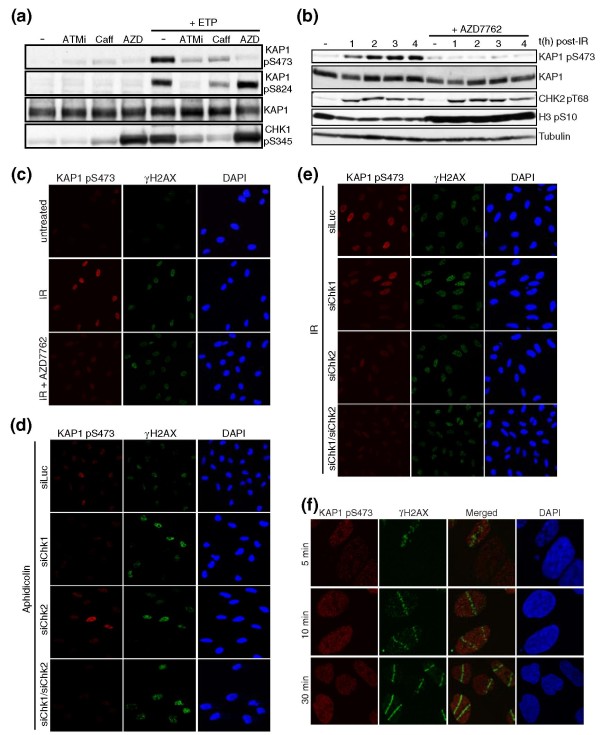
**KAP1 phospho-Ser473 after DNA damage is Chk1- and Chk2-dependent**. **(a) **Etoposide-induced KAP1 Ser473 phosphorylation is abolished by Chk1/Chk2 inhibition and reduced upon ATM inhibition. U2OS cells were untreated or treated with 5 μM etoposide (ETP) for 4 h in the presence or absence of KU55933 (ATMi), caffeine (Caff), or AZD7762 (AZD). **(b) **KAP1 phospho-Ser473 induction after 20 Gy of IR is abolished by AZD7762 (the drug was not removed during the recovery time). Chk2 phospho-Thr68 was used as readout of DNA damage and histone H3 phospho-Ser10 was used as readout for the G2/M checkpoint. **(c) **AZD7762 decreases KAP1 phospho-Ser473 on immunofluorescence; U2OS cells were treated as in (b). **(d) **KAP1 Ser473 is targeted by Chk1. U2OS cells were transfected with either siLuc, siChk1, siChk2, or both siChk1 and siChk2, then treated with 10 μM aphidicolin for 1 h. **(e) **KAP1 Ser473 is targeted by Chk2. U2OS cells were transfected as in (d) and treated as in (b). **(f) **KAP1 phospho-Ser473 is neither recruited nor excluded from laser-induced DNA-damage sites. Cells were fixed 5, 10 or 30 minutes after micro-irradiation.

To explore the factor-dependencies of KAP1 Ser473 phosphorylation, we carried out experiments with the selective Chk1/Chk2 inhibitor AZD7762 [35], the specific ATM inhibitor KU55933 [36], or caffeine at a concentration that inhibits both ATM and ATR [37]. This revealed that phosphorylation of KAP1 Ser473 in response to etoposide or IR was essentially abolished when cells were incubated with AZD7762, indicating that KAP1 Ser473 is a Chk1/2 target (Figures 4a-c). By contrast, and consistent with our data indicating that phosphorylation of KAP1 Ser473 and Ser824 operate independently (Figure 3d), Chk1/2 inhibition by AZD7762 did not diminish KAP1 Ser824 phosphorylation, which was only decreased upon ATM inhibition (Figure 4a). Furthermore, KAP1 Ser473 phosphorylation was reduced by caffeine and KU55933, in line with Chk1 being targeted by ATR in response to etoposide treatment in a manner that is promoted by ATM [38] (Figure 4a; note that Chk1 Ser345 phosphorylation upon etoposide treatment was also inhibited by caffeine and by ATM inhibition). Similar to the effects observed for etoposide, IR-induced KAP1 Ser473 phosphorylation was also virtually abolished by AZD7762 treatment (Figure 4b). As expected, AZD7762 did not prevent ATM-mediated phosphorylation of Chk2 on Thr68 but, in line with the known checkpoint functions of Chk1, it abrogated DNA-damage-induced G2/M cell cycle arrest, as evidenced by it preventing the diminution of mitotic histone H3 Ser10 phosphorylation upon IR treatment (Figure 4b).

Because AZD7762 inhibits both Chk1 and Chk2 [35], and as previous work has indicated that Chk1 and Chk2 have overlapping substrate specificities [39], we employed siRNA-depletion methods to determine whether both Chk1 and Chk2 can target KAP1 Ser473. As shown in Figure 4d, Chk1 depletion but not Chk2 depletion abolished KAP1 Ser473 phosphorylation induced by aphidicolin, which inhibits replicative DNA polymerases and activates the ATR/Chk1 pathway in S-phase cells [40] (note that γH2AX staining indicates that DNA damage still occurred in Chk1-depleted cells). By contrast, when we induced DNA damage by IR, KAP1 Ser473 phosphorylation was only reduced slightly by Chk1 depletion but was reduced much more substantially upon Chk2 depletion (Figure 4e; note that full abrogation of KAP1 Ser473 phosphorylation after IR required co-depletion of Chk1 and Chk2). These results therefore indicated that both Chk1 and Chk2 can target KAP1 Ser473, and are in agreement with IR triggering both the ATM/Chk2 and ATR/Chk1 pathways [38].

Various proteins involved in DNA-damage signaling and repair form discrete nuclear foci upon IR, marking sites where DNA damage has occurred [41]. This is not the case, however, for KAP1 or KAP1 phospho-Ser824, which are evenly distributed throughout the nucleoplasm after DNA damage [31]. Similarly, we observed pan-nuclear staining with the KAP1 phospho-Ser473 antibody (Figures 3c and 4c-e). To provide a more detailed analysis of Ser473 phosphorylation dynamics, we used laser micro-irradiation to induce localized DNA damage [41]. While such an approach has shown that KAP1 is transiently recruited to sites of damage, where it is phosphorylated on Ser824 and then released [31], we observed neither association nor exclusion of KAP1 phospho-Ser473 from sites of laser micro-irradiation (Figure 4f). These data suggested that KAP1 Ser473 phosphorylation by Chk1 and Chk2 does not take place predominantly at sites of DNA damage, and are consistent with previous work indicating that, following their DNA-damage-localized phosphorylation and activation by ATR and ATM, Chk1 and Chk2 dissociate from chromatin to phosphorylate their substrates [42,43].

We carried out various functional studies to ascribe a specific function to KAP1 Ser473. For example, we found that mutating Ser473 did not affect KAP1 phosphorylation on Ser824 (Figure 3d) or KAP1 SUMOylation (Additional file 3), which has been implicated in transcriptional silencing [44]. Furthermore, in line with previous findings [31], we found that DNA damage did not perceptibly change KAP1 interactions with its binding partners SETDB1, HDAC1 and MDM2 (Additional file 4). Importantly, we discovered that the recently reported serum induction of KAP1 Ser473 phosphorylation [45] was not affected by AZD7762 (Figure S4a in Additional file 5), indicating that another kinase(s) targets this site upon serum stimulation. In line with this and the fact that we observed similar levels of IR-induced KAP1 Ser473 phosphorylation in all cells of an asynchronously growing population (Figures 3c and 4c), we found no correlation between DNA-damage-induced KAP1 Ser473 phosphorylation and cell-cycle stage (Figure S4b in Additional file 5). Moreover, although a recent report [45] concluded that cell-cycle regulated KAP1 phosphorylation on Ser473 controls the interaction between KAP1 and HP1β, we observed no effect of mutating Ser473 on the binding of KAP1 to HP1 (Figure S4c in Additional file 5; as shown in Figure S4d in Additional file 5 there was also no apparent relationship between KAP1 Ser473 phosphorylation and chromatin status). We therefore conclude that the effects of Ser473 phosphorylation are too subtle to be detected by existing assays, or that this phosphorylation site regulates as yet undefined KAP1 functions.

## Discussion

We have used a chemical genetics approach, employing a mutated *as*-Chk1 derivative that can utilize the ATP analogue N6B-ATPγS, to identify proteins that can serve as direct substrates for Chk1. Through defining a considerable number of Chk1 phosphorylation sites using this technique, we have further refined the Chk1 consensus sequence. Strikingly, our analyses indicate that, in addition to the over-representation of certain amino acid residues at particular positions within the Chk1 target motif, there are also other residues that are markedly under-represented in certain positions. Thus, we are led to the overall target consensus motif for Chk1 being R/K-R/K-d/e-t-S*/T*-X-r/k-r, where capital and lower-case letters reflect selection and counter-selection, respectively. Notably, through further investigations into various subsets of Chk1 targets, we have found that the 'rules' for Chk1 target recognition cannot be explained simply on the basis of selecting or counter-selecting for certain residues at specific positions. Instead, more complex, context-dependent selections also seem to operate, and it appears that more than one class of target motif may exist, perhaps pointing towards Chk1 using adaptor proteins to recognize its substrates. It should be possible to explore these ideas by mutational analyses and by structural studies of Chk1 in association with various types of target sequence, and it will be intriguing to see whether similar situations exist for other protein kinases.

In addition to identifying and validating KAP1 as a Chk1 target, our screen identified several other proteins involved in DNA replication and repair, including Fen1, Rif1, TICRR/Treslin and Ku70 (Table 1). It will be interesting, therefore, to investigate the potential effects of Chk1 on the activities of such factors. Notably, however, a considerable proportion of the Chk1 substrates we identified have been assigned roles in transcription and/or RNA processing, cellular functions that are being increasingly linked to the control of genome stability [46]. In line with this, we found that several of the newly identified Chk1 substrates functionally clustered around transcription factor ZNF143, which is known to control expression of DNA repair- and cell-cycle-related genes [47,48], and around SARNP, a protein linked to transcription and RNA export with a suggested role in cell growth and carcinogenesis [49,50] (Additional file 6). Further work will be required to validate such factors as true Chk1 substrates and determine whether and how Chk1 - and possibly Chk2 and MK2, which have similar consensus motifs to Chk1 [4] - regulate the events that they control. Finally, we note that, because Chk1 inhibitors are being assessed as anti-cancer agents [51], understanding the repertoire and functional consequences of Chk1-mediated phosphorylations might suggest how Chk1 inhibitors can be best exploited clinically. In order to most effectively develop Chk1 inhibitors, it will be necessary to have a robust and accurate readout of Chk1 activity. While previous work has mainly used phosphorylation of Chk1 itself on Ser345 as a biomarker for Chk1 inhibition, there are two limitations to this: first, Chk1 Ser345 phosphorylation is only clearly detected after prolonged treatments with Chk1 inhibitors; and second, Ser345 phosphorylation is an indirect readout of Chk1 inhibition as it appears to measure the hyper-activation of ATR that occurs when Chk1 is inhibited [52]. Our work highlights the potential for measuring KAP1 Ser473 phosphorylation as an alternative, more direct way of monitoring Chk1 activity and its inhibition.

## Conclusions

We have described the results of a screen for novel Chk1 substrates. The approach used employed an analogue-sensitive mutant of Chk1 that can directly label substrates in cell extracts by it using a thio-phosphate-bearing ATP analogue. Thus, we have identified 268 phosphorylation sites in 171 proteins. Based on these results, we have refined the preferred Chk1 target phosphorylation motif. Furthermore, as proof-of-concept for the screening approach, we established that one of the sites identified, Ser473 on the transcriptional co-repressor KAP1, indeed serves as a DNA-damage-responsive Chk1/Chk2 target in cells. In addition to providing clues into how Chk1 may control diverse cellular functions and defining a marker of potential utility in evaluating the effects of Chk1 inhibitors *in vivo*, our data provide additional resources that should be valuable for future research.

## Materials and methods

### DNA constructs and transfections

pEGFP-HA-KAP1wt (wild type) and pEGFP-HA-KAP1S824A were a gift from Y Shiloh (Tel Aviv University, Israel). pEGFP-HA-KAP1S473A and pEGFP-HA-KAP1S473D were made by site-directed mutagenesis of pEGFP-HA-KAP1wt using the primers: KAP1-S473A-F, 5'-GAAACGGTCCCGCGCAGGTGAGGGCGAG-3'; KAP1-S473A-R, 5'-CTCGCCCTCACCTGCGCGGGACCGTTTC-3'; KAP1-S473D-F, 5'-GGTGTGAAACGGTCCCGCGACGGTGAGGGCGAGGTGAGC-3'; KAP1-S473D-R, 5'-GCTCACCTCGCCCTCACCGTCGCGGGACCGTTTCACACC-3'.

Plasmid DNA was transfected with FuGENE 6 reagent (Roche Diagnostics Ltd., Burgess Hill, UK)) following the manufacturer's instructions.

### Expression and purification of recombinant proteins

pFastBac-TEV-SBP-Chk1wt was prepared by amplifying Chk1 from pCIneo-FLAG-Chk1 (provided by J Bartek, Institute of Cancer Biology, Copenhagen, Denmark) and cloning it into pFastBac1-TEV-SBP (gift from P Marco-Casanova, Gurdon Institute, Cambridge, UK) via EcoRI and XbaI restriction sites. Bacmids were prepared in DH10Bac™ *Escherichia coli *cells (Invitrogen, Carlsbad, CA, USA)) following the manufacturer's protocol. Primers for site-directed mutagenesis of Chk1 Leu84 were: Chk1L84G-F, 5'-GCAATATCCAATATTTATTTGGGGAGTACTGTAGTGGAGGAGAGC-3'; Chk1L84G-R, 5'-GCTCTCCTCCACTACAGTACTCCCCAAATAAATATTGGATATTGC-3'; Chk1L84A-F, 5'-GCAATATCCAATATTTATTTGCGGAGTACTGTAGTGGAGGAGAGC-3'; and Chk1L84A-R, 5'-GCTCTCCTCCACTACAGTACTCCGCAAATAAATATTGGATATTGC-3'. SBP-tagged wild type and mutated Chk1 proteins were expressed in Sf9 insect cells and purified to homogeneity as described for SBP-tag purification [53]. pGEX20T-Cdc25A was a gift from J Bartek (Institute of Cancer Biology, Copenhagen, Denmark). GST-Cdc25A was expressed in BL21 *E. coli *cells and purified with glutathione sepharose beads following the manufacturer's instructions.

### Protein kinase assays

All *in vitro *kinase assays were done in Chk1 kinase buffer (50 mM HEPES, pH 7.4; 13.5 mM MgCl_2_; and 1 mM dithiothreitol) in the presence of 1 mM Na_3_VO_4 _and 1 mM ATP or ATP analogue. Reactions were incubated for 30 minutes at 30°C and stopped by addition of 10 mM EDTA, pH 8. For western blotting, proteins were mixed with Laemmli buffer and separated on 9% SDS-polyacrylamide gels.

### Western blotting

Proteins were separated by SDS-PAGE. Antibodies used were: Chk1 (1:100 mouse G4; Santa Cruz Biotechnology, Inc., Santa Cruz, CA, USA), Chk1 phospho-Ser317 (1:1,000 rabbit; Cell Signaling Technology, Danvers, MA, USA), Chk1 phospho-Ser345 (1:5,000 rabbit; Cell Signaling), Chk2 phospho-Thr-68 (1:1,000 rabbit; Cell Signaling), Cdc25A (1:100 mouse; Santa Cruz), Cdc25A phospho-Ser123 was provided by E Appella (National Cancer Institute, Bethesda, US), GFP (1:1,000 mouse; Roche), histone H3 phospho-Ser10 (1:5,000 mouse; Abcam, Cambridge, UK), KAP1 (1:500 rabbit; Santa Cruz), KAP1 phospho-Ser824 (1:1,000 rabbit; Bethyl Laboratories, Inc., Montgomery, TX, USA), KAP1 phospho-Ser473 (1:1,000 rabbit; BioLegend), tubulin (1:5,000 mouse; Sigma Aldrich Company, Ltd., Dorset, UK), thiophosphate-ester-specific antibody (1:5,000; Epitomics, Inc., Burlingame, CA, USA) according to the manufacturers' instructions.

### Large-scale kinase assay, purification of phospho-peptides and mass spectrometry

Large-scale Chk1 kinase assay and subsequent peptide enrichment was as previously described [54]. Briefly, 1 mg of HeLa nuclear extract (Cil Biotech, Mons, Belgium) was incubated with 10 μg of SBP-Chk1L84G in the presence of 1 mM Na_3_VO_4 _and 1 mM N6B-ATPγS in 1× Chk1 kinase buffer for 30 minutes at 30°C. Reactions were stopped by addition of EDTA. Trypsin digestion was done in denaturing buffer following a standard protocol. Phosphopeptides were enriched using a previously described method [16]. Briefly, 100 μl of iodoacetyl-agarose beads (SulfoLink gel, Thermo Fisher Scientific Inc., Rockford, IL, USA) in 100 μl of 50% acetonitrile were added to trypsin-digested peptides. The beads were extensively washed with 2 ml each of water, 5 M NaCl, 50% acetonitrile, and 5% formic acid in water, sequentially. Phosphopeptides were eluted using 200 μl of a 1 mg/ml solution of Oxone, and purified on C18 StageTips [55]. Phosphopeptides were analyzed on a linear ion trap/Orbitrap mass spectrometer (LTQ-Orbitrap XL), as described previously [56]. Raw MS data were processed using MaxQuant [57]. Data were searched using the Mascot search engine (Matrix Science Ltd., London, UK), and peptides were identified using MaxQuant at a false discovery rate of 1% for peptides and proteins. Cysteine carbamidomethylation was searched as a fixed modification, whereas amino-terminal protein acetylation, phosphorylation of Ser, Thr, and Tyr, and oxidation of Met were searched as variable modifications. Raw MS data are available at the PeptideAtlas repository [58].

### Cell culture and reagents

U2OS cells were used throughout and grown in DMEM supplemented with 10% fetal bovine serum, penicillin, streptomycin, and glutamine. Stable clones expressing GFP-KAP1 were selected adding G-418 (0.5 mg/ml) to the medium. Aphidicolin, caffeine, etoposide, hydroxyurea and camptothecin were from Sigma-Aldrich; phleomycin was from Melford Laboratories Ltd., Ipswich, UK. IR was applied with a Faxitron X-ray cabinet. UV irradiation was done on cells covered in 1× PBS at a rate of 0.7 J/m^2 ^per second. AZD7762 was provided by AstraZeneca and used at 50 nM. KU55933 [36] was used at 20 μM. Caffeine was used at 4 mM. All incubations with inhibitors started 1 h before any other treatment was applied. N-6-Benzyladenosine-5'-O-triphosphate (N6B-ATP) and N-6-benzyladenosine-5'-O-(3-thiotriphosphate) (N6B-ATPγS) were from BIOLOG Life Science Institute Forschungslabor und Biochemica-Vertrieb GmbH, Bremen, Germany.

### siRNAs and transfections

siChk1 and siChk2 were with siGENOME SMARTpool siRNA (Thermo Fisher Scientific Dharmacon Products, Lafayette, CO, USA); siLuc (5'-cguacgcggaauacuucgatt-3') and siKAP1 [31] were from Eurofins MWG Operon, Ebersberg, Germany. Transfections were done with Lipofectamine RNAiMAX (Invitrogen). Cells were treated 12 h (siChk1 and siChk2) or 48 h (siKAP1) afterwards.

### Immunofluorescence

Cells were grown on poly-L-lysine-coated coverslips, fixed with 2% paraformaldehyde for 10 minutes and permeabilized with 1× PBS containing 0.2% (v/v) Triton X-100 for 5 minutes. Primary antibody staining was for 1 h in 5% fetal bovine serum in 1× PBS with KAP1 phospho-Ser473 (1:100 rabbit; BioLegend) and γH2AX (1:1,000 mouse; Millipore, Billerica, MA, USA). Secondary antibody staining was with goat anti-mouse Alexa Fluor 488 or goat anti-rabbit Alexa Fluor 594 (1:1,000; Invitrogen, Carlsbad, CA, USA) for 30 minutes. Coverslips were washed three times with 1× PBS and mounted on slides with Vectashield solution (Vector Laboratories Ltd., Peterborough, UK) containing 4',6-diamidino-2-phenylindole (DAPI) to stain DNA. All incubations were done at room temperature.

### Laser micro-irradiation and cell imaging

For generation of localized damage in cellular DNA by exposure to a UV-A laser beam [43,59], cells were plated on glass-bottomed dishes (WillCo Wells B.V., Amsterdam, Netherlands) and pre-sensitized with 10 μM 5-bromo-2'-deoxyuridine (BrdU; Sigma-Aldrich) in phenol-red-free medium (Invitrogen) for 24 h at 37°C. Laser micro-irradiation was done by using a FluoView 1000 confocal microscope (Olympus) equipped with a 37°C heating stage (ibidi GmbH, Martinsried, Germany) and a 405 nm laser diode (6 mW) focused through a 60× UPlanSApo/1.35 oil objective to yield a spot size of 0.5 to 1 mm. Time of cell exposure to the laser beam was around 250 ms (fast scanning mode). Laser settings (0.40 mW output, 50 scans) were chosen that generate a DDR restricted to the laser path in a pre-sensitization-dependent manner without noticeable cytotoxicity.

## Abbreviations

*as*-kinase: analogue-sensitive kinase; ATM: ataxia-telangiectasia mutated; ATR: ATM and Rad3 related; DDR: DNA-damage response; DSB: double-strand break; GFP: green fluorescent protein; HP1: heterochromatin protein 1; IR: ionizing radiation; KAP1: Krüppel-associated box domain-associated protein 1; MK2: p38MAPK/MAPKAP-K2; N6B-ATP: N6-benzyl ATP; PBS: phosphate-buffered saline; siRNA: short-interfering RNA.

## Competing interests

The authors declare that they have no competing interests.

## Authors' contributions

MB and JVF conceived the study and wrote the manuscript, prepared and tested the *as*-Chk1 kinase, performed the kinase assay and validated KAP1 as a substrate. SPJ conceived the study and wrote the manuscript. NT performed tissue culture and immunoprecipitation experiments. SAW and CC purified the modified peptides, performed the mass spectrometry and identified the phosphorylated residues. All authors have read and approved the final version of the manuscript.

## Supplementary Material

Additional file 1 **Table S1 - Excel sheet showing a complete list of identified peptides and phospho-sites**. Raw MS data are available at the PeptideAtlas repository [58]. Click here for file

Additional file 2 **Figure S1 - frequencies of amino acids surrounding phospho-Thr (left panel) or phospho-Ser (right panel) on peptides containing a basic residue (Arg or Lys) at position -3 identified in our screen**. See legend of Figure 2 in main text for details. Click here for file

Additional file 3 **Figure S2 - mutation of KAP1 Ser-473 does not affect KAP1 SUMOylation**. SUMOylated proteins were immunoprecipitated from U2OS cells expressing RFP-SUMO1 and GFP-KAP1 versions, and western blots were probed to detect SUMOylated GFP-KAP1. Click here for file

Additional file 4 **Figure S3 - DNA damage does not affect the interaction between KAP1 and SETDB1, HDAC1, or MDM2**. HEK293 cells were transfected with GFP-KAP1 and treated with 5 **μ**M etoposide (ETP) for 4 h in the presence or absence of 20 **μ**M KU55933 (ATMi) or 50 nM AZD7762 (AZD). GFP-KAP1 was immunoprecipitated and interaction with SETDB1, HDAC1, and MDM2 was checked on western blot. KAP1 phospho-Ser473 was used as readout for both DNA-damage induction and ATM and Chk1/Chk2 inhibition. Click here for file

Additional file 5 **Figure S4 - cell-cycle phosphorylation of KAP1 Ser-473 is Chk1/Chk2 independent**. **(a) **KAP1 phospho-Ser473 upon serum addition is insensitive to the Chk1/Chk2 inhibitor AZD7762. RPE-1 cells were serum-starved for 48 h and then released in medium containing 20% serum for the indicated times in the presence or absence of 50 nM AZD7762. Chk1 phospho-Ser345 was used as readout for AZD7762. **(b) **KAP1 phospho-Ser473 shows no correlation with cyclin A staining after DNA damage. RPE-1 cells were treated with 20 Gy ionizing radiation (IR) and fixed 2 h afterwards. **(c) **KAP1 Ser473 mutants show no difference on the interaction with heterochromatin protein 1β (HP1β). Cell extracts from U2OS cells expressing GFP-KAP1 versions were subjected to pull-downs with recombinant GST- HP1β. **(d) **KAP1 phospho-Ser473 does not preferentially co-localize with heterochromatic regions. Mouse embryonic fibroblasts were treated as in (b). Heterochromatin is detected as DAPI-dense regions. γH2AX was used to assess DNA damage. Click here for file

Additional file 6 **Figure S5 - novel Chk1 substrates cluster around proteins involved in RNA metabolism**. Proteins identified in this screen are labeled in red. A solid line between two proteins indicates a direct interaction; an arrow indicates that protein A acts on protein B. Protein clusters were identified using Ingenuity software (Ingenuity Systems, Inc., Redwood City, CA, USA). Click here for file

Additional file 7 **Supplementary materials and methods**. PDF describing materials and methods used for Additional files 2 to 6. Click here for file
