# Supplementary figures and images for: A phospho-proteomic screen identifies substrates of the checkpoint kinase Chk1
Source: Genome Biol. 2011 Aug 18;12(8):R78. doi: 10.1186/gb-2011-12-8-r78 (PMC3245618; doi:10.1186/gb-2011-12-8-r78)

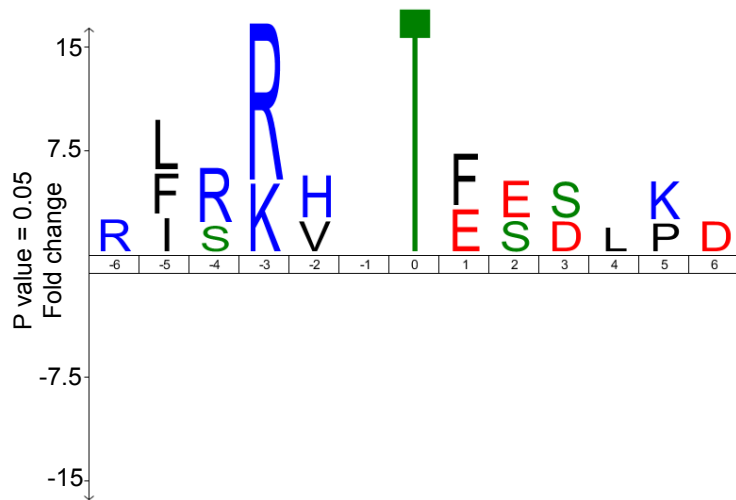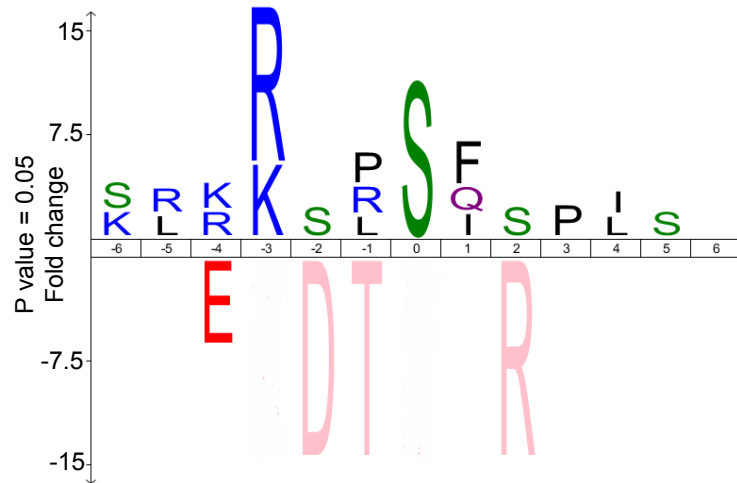

Blasius et al Supplementary Figure S1

Supplement: Additional file 2 — Figure S1 - frequencies of amino acids surrounding phospho-Thr (left panel) or phospho-Ser (right panel) on peptides containing a basic residue (Arg or Lys) at position -3 identified in our screen. See legend of Figure 2 in main text for details. [file gb-2011-12-8-r78-S2.PDF]

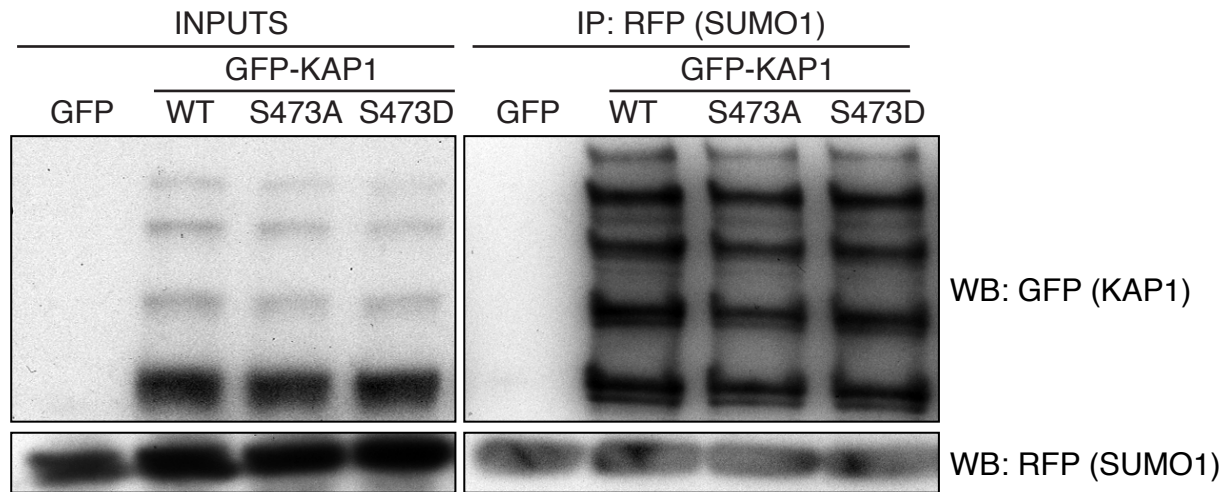

Blasius et al Supplementary Figure S2

Supplement: Additional file 3 — Figure S2 - mutation of KAP1 Ser-473 does not affect KAP1 SUMOylation. SUMOylated proteins were immunoprecipitated from U2OS cells expressing RFP-SUMO1 and GFP-KAP1 versions, and western blots were probed to detect SUMOylated GFP-KAP1. [file gb-2011-12-8-r78-S3.PDF]

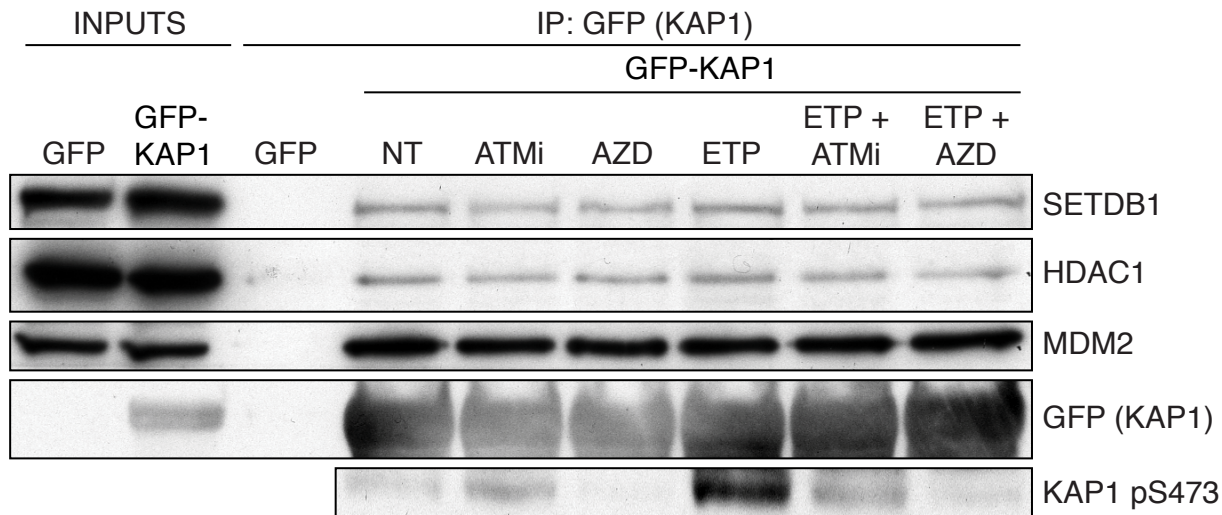

Blasius et al Supplementary Figure S3

Supplement: Additional file 4 — Figure S3 - DNA damage does not affect the interaction between KAP1 and SETDB1, HDAC1, or MDM2. HEK293 cells were transfected with GFP-KAP1 and treated with 5 μM etoposide (ETP) for 4 h in the presence or absence of 20 μM KU55933 (ATMi) or 50 nM AZD7762 (AZD). GFP-KAP1 was immunoprecipitated and interaction with SETDB1, HDAC1, and MDM2 was checked on western blot. KAP1 phospho-Ser473 was used as readout for both DNA-damage induction and ATM and Chk1/Chk2 inhibition. [file gb-2011-12-8-r78-S4.PDF]

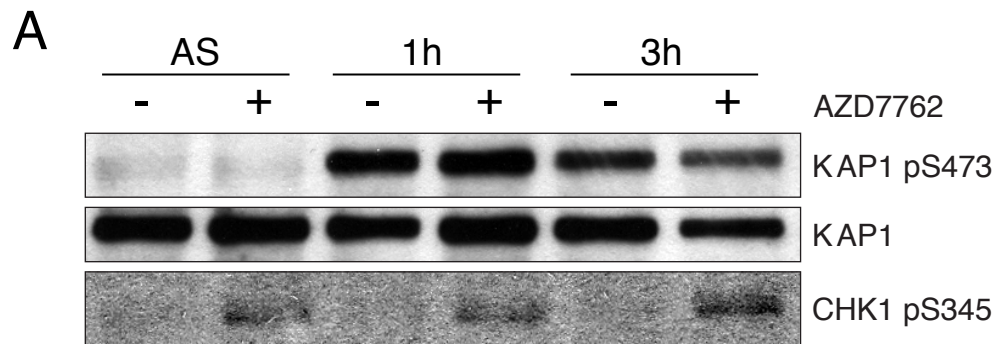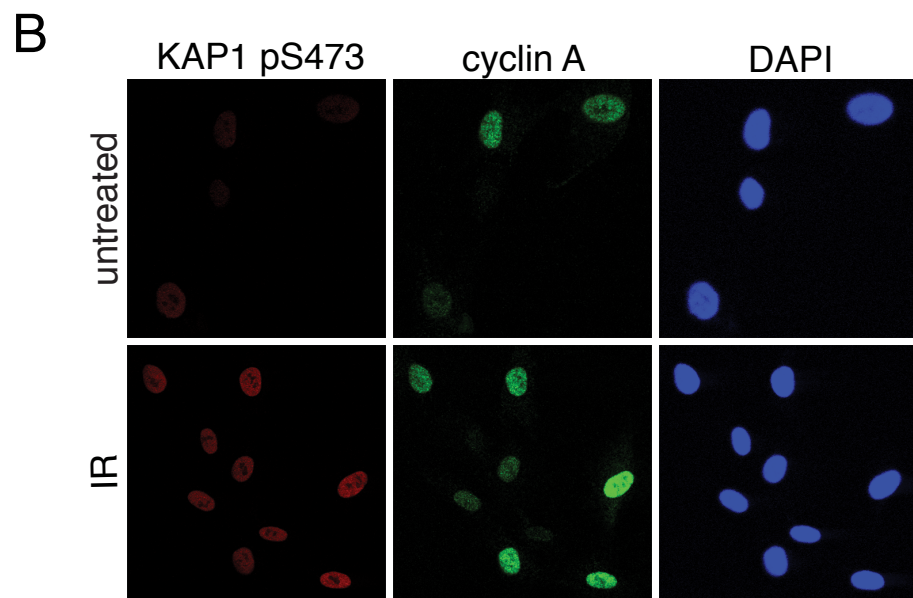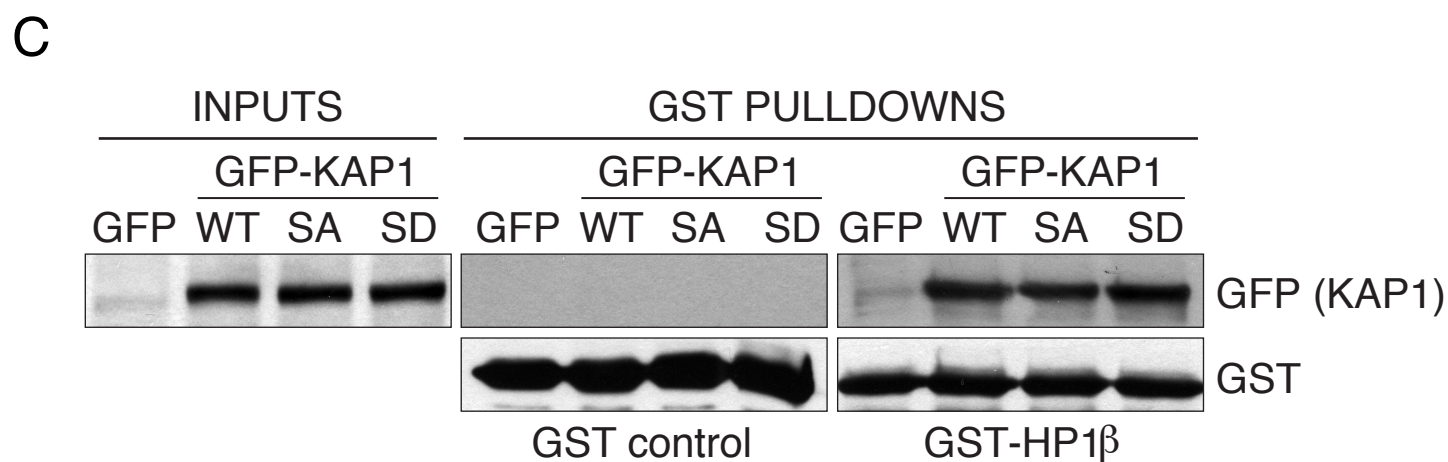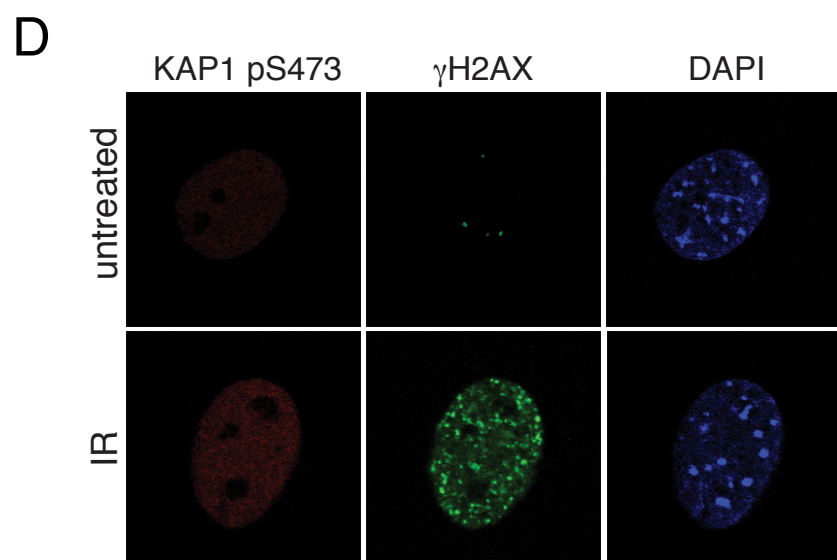

Supplement: Additional file 5 — Figure S4 - cell-cycle phosphorylation of KAP1 Ser-473 is Chk1/Chk2 independent. (a) KAP1 phospho-Ser473 upon serum addition is insensitive to the Chk1/Chk2 inhibitor AZD7762. RPE-1 cells were serum-starved for 48 h and then released in medium containing 20% serum for the indicated times in the presence or absence of 50 nM AZD7762. Chk1 phospho-Ser345 was used as readout for AZD7762. (b) KAP1 phospho-Ser473 shows no correlation with cyclin A staining after DNA damage. RPE-1 cells were treated with 20 Gy ionizing radiation (IR) and fixed 2 h afterwards. (c) KAP1 Ser473 mutants show no difference on the interaction with heterochromatin protein 1β (HP1β). Cell extracts from U2OS cells expressing GFP-KAP1 versions were subjected to pull-downs with recombinant GST- HP1β. (d) KAP1 phospho-Ser473 does not preferentially co-localize with heterochromatic regions. Mouse embryonic fibroblasts were treated as in (b). Heterochromatin is detected as DAPI-dense regions. γH2AX was used to assess DNA damage. [file gb-2011-12-8-r78-S5.PDF]

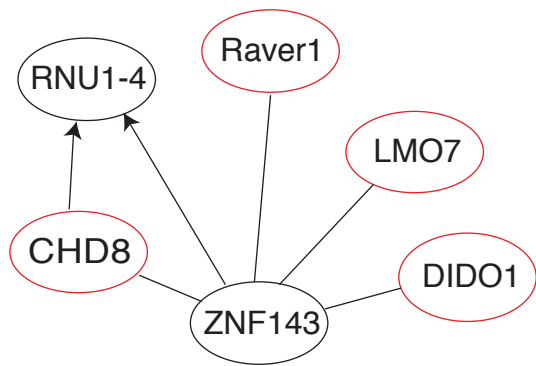

Cluster 1

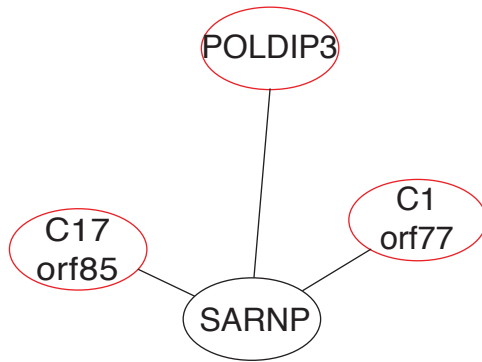

Cluster 2

Blasius et al Supplementary Figure S5

Supplement: Additional file 6 — Figure S5 - novel Chk1 substrates cluster around proteins involved in RNA metabolism. Proteins identified in this screen are labeled in red. A solid line between two proteins indicates a direct interaction; an arrow indicates that protein A acts on protein B. Protein clusters were identified using Ingenuity software (Ingenuity Systems, Inc., Redwood City, CA, USA). [file gb-2011-12-8-r78-S6.PDF]
