# Supplementary material for: A phospho-proteomic screen identifies substrates of the checkpoint kinase Chk1
Source: Genome Biol. 2011 Aug 18;12(8):R78. doi: 10.1186/gb-2011-12-8-r78 (PMC3245618; doi:10.1186/gb-2011-12-8-r78)
Supplement: Additional file 7 — Supplementary materials and methods. PDF describing materials and methods used for Additional files 2 to 6. [file gb-2011-12-8-r78-S7.PDF]

Additional file 7

A phospho-proteomic screen identifies substrates of the checkpoint kinase Chk1

Melanie Blasius, Josep V. Forment, Neha Thakkar, Sebastian A. Wagner, Chunaram Choudhary, and Stephen P. Jackson

Additional file 7: File including supplementary materials and methods for Additional files 2-6, figures S2-S6.

Materials and methods

Cell culture

Human HEK293 cells and mouse embryonic fibroblasts (MEFs) were cultured in DMEM supplemented with 10% fetal bovine serum (FBS), penicillin, streptomycin, and glutamine. Human RPE-1 hTERT cells were cultured in DMEM F-12 Ham supplemented with 10% fetal bovine serum (FBS), sodium bicarbonate, penicillin, streptomycin, and glutamine.

Antibodies

For western blot: SETDB1 (rabbit 1:2000; Millipore), RFP (rabbit 1:1000; Abcam), HDAC1 (rabbit 1:3000; Abcam), MDM2 (mouse 1:200; Oncogene), GST (1:1000; Santa Cruz). For immunofluorescence: Cyclin A (mouse 1:100, BD Biosciences).

Immunoprecipitations

Cell pellets from U2OS cells stably expressing GFP-KAP1 were resuspended in SUMO lysis buffer containing 20 mM HEPES pH 7.4, 500 mM NaCl, 1.5 mM $MgCl_2$, 1 mM EGTA, 1% Tween-20, 5% glycerol, serine/threonine phosphatase inhibitors, protease inhibitor cocktail (Roche) and 10 mM N-ethylmaleimide (Sigma-Aldrich), and incubated 30 min on ice. Extracts were cleared by centrifugation at 14,000 rpm for 30 min at 4°C and then diluted to 150 mM NaCl. 1 mg of protein extract was incubated with GFP-trap beads (GFP-Trap_A, Chromotek) for 2 h at 4°C on a rolling wheel. Beads were washed thrice with lysis buffer containing 150 mM NaCl, and then resuspended in 2x Laemmli buffer, boiled for 5 min, and loaded on SDS-PAGE.

KAP1 SUMOylation

Human HEK293 cells were transfected with pEGFP-C1 (Clontech), pRFP-SUMO1 (kindly provided by Y. Galanty), and/or pEGFP-HA-KAP1 versions. Cell extracts were prepared in SUMO lysis buffer. Extracts were then sonicated, cleared by centrifugation at 14,000 rpm for 30 min at 4°C, and then diluted 1:2 with the same buffer lacking NaCl to leave the final salt concentration at 250 mM. 1 mg of protein extract was incubated with RFP-trap beads (RFP-Trap_A, Chromotek) for 2 h at 4°C on a rolling wheel. Beads were washed twice with SUMO lysis buffer containing 500 mM NaCl, twice with SUMO lysis buffer containing 250 mM NaCl, and then resuspended in 2x Laemmli buffer, boiled for 5 min, and loaded on SDS-PAGE.

GST pulldowns

GST-HP1α, GST-HP1β and GST-HP1γ were a generous gift of T. Bartke (Gurdon Institute, Cambridge, UK). 10 μg of purified GST-HP1 protein were bound to

Glutathione sepharose beads (GE Healthcare Life Sciences), washed in phosphate-buffered saline (PBS) and incubated with 1 mg of total cell extract for 2 h at 4°C. Beads were washed 5 times with pull-down buffer (20 mM HEPES, 150 mM NaCl, 0.5 mM EDTA, 10% glycerol, 0.05% Triton X-100 and 1 mM DTT), and bound proteins were eluted in Laemmli buffer.
